# Thermal and Nutritional Regulation of Ribosome Hibernation in Staphylococcus aureus

**DOI:** 10.1128/JB.00426-18

**Published:** 2018-11-26

**Authors:** Arnab Basu, Kathryn E. Shields, Christopher S. Eickhoff, Daniel F. Hoft, Mee-Ngan F. Yap

**Affiliations:** a Edward A. Doisy Department of Biochemistry and Molecular Biology, Saint Louis University School of Medicine, Saint Louis, Missouri, USA; b Division of Infectious Diseases, Allergy and Immunology, Department of Internal Medicine, Saint Louis University School of Medicine, Saint Louis, Missouri, USA; Ohio State University

**Keywords:** HPF, ribosome, HflX, CodY, general stress response, hibernation

## Abstract

The translationally silent 100S ribosome is a poorly understood form of the dimeric 70S complex that is ubiquitously found in all bacterial phyla. The elimination of the hibernating 100S ribosome leads to translational derepression, ribosome instability, antibiotic sensitivity, and biofilm defects in some bacteria. In *Firmicutes*, such as the opportunistic pathogen Staphylococcus aureus, a 190-amino acid protein called hibernating-promoting factor (HPF) dimerizes and conjoins two 70S ribosomes through a direct interaction between the HPF homodimer, with each HPF monomer tethered on an individual 70S complex. While the formation of the 100S ribosome in gammaproteobacteria and cyanobacteria is exclusively induced during postexponential growth phase and darkness, respectively, the 100S ribosomes in *Firmicutes* are constitutively produced from the lag-logarithmic phase through the post-stationary phase. Very little is known about the regulatory pathways that control *hpf* expression and 100S ribosome abundance. Here, we show that a general stress response (GSR) sigma factor (SigB) and a GTP-sensing transcription factor (CodY) integrate nutrient and thermal signals to regulate *hpf* synthesis in S. aureus, resulting in an enhanced virulence of the pathogen in a mouse model of septicemic infection. CodY-dependent regulation of *hpf* is strain specific. An epistasis analysis further demonstrated that CodY functions upstream of the GSR pathway in a condition-dependent manner. The results reveal an important link between S. aureus stress physiology, ribosome metabolism, and infection biology.

**IMPORTANCE** The dimerization of 70S ribosomes (100S complex) plays an important role in translational regulation and infectivity of the major human pathogen Staphylococcus aureus. Although the dimerizing factor HPF has been characterized biochemically, the pathways that regulate 100S ribosome abundance remain elusive. We identified a metabolite- and nutrient-sensing transcription factor, CodY, that serves both as an activator and a repressor of *hpf* expression in nutrient- and temperature-dependent manners. Furthermore, CodY-mediated activation of *hpf* masks a secondary *hpf* transcript derived from a general stress response SigB promoter. CodY and SigB regulate a repertoire of virulence genes. The unexpected link between ribosome homeostasis and the two master virulence regulators provides new opportunities for alternative druggable sites.

## Abstract (precis)

The dimerization of 70S ribosomes (100S complex) plays an important role in translational regulation and infectivity of the major human pathogen Staphylococcus aureus. Although the dimerizing factor HPF has been characterized biochemically, the pathways that regulate 100S ribosome abundance remain elusive. We identified a metabolite- and nutrient-sensing transcription factor, CodY, that serves both as an activator and a repressor of *hpf* expression in nutrient- and temperature-dependent manners. Furthermore, CodY-mediated activation of *hpf* masks a secondary *hpf* transcript derived from a general stress response SigB promoter. CodY and SigB regulate a repertoire of virulence genes. The unexpected link between ribosome homeostasis and the two master virulence regulators provides new opportunities for alternative druggable sites.

## INTRODUCTION

The robustness of bacterial growth under conditions that favor proliferation is fine-tuned to ribosome synthesis and translational efficiency. Conversely, ribosome production is constrained in slow-growing or dormant cells. The maintenance of the integrity of the existing ribosomes and the ability to resume translation are critical for the resuscitation from unfavorable environments (1–6). To preserve a sufficient ribosome pool for regrowth without energetically costly translation, vacant 70S ribosomes self-dimerize to form the inactive hibernating 100S ribosome. Ribosome hibernation is required for bacterial survival *in vitro*, which has been linked to a reduced ribosome degradation, the suppression of superfluous translation, enhanced antibiotic and stress tolerance, and biofilm formation (3–5, 7–15). For reviews see references 16 and 17.

In gammaproteobacteria, including Escherichia coli (11, 18, 19), vibrios (14), and pseudomonads (4), two small ribosome-binding proteins (RMF and HPF_short_) concertedly induce the formation of the 100S complex. A third ribosome-silencing factor, YfiA (also known as pY or RaiA), exists only in some gammaproteobacteria and plant chloroplasts (named PSRP1). YfiA binds and inactivates the 70S ribosome without 70S dimerization (20–23). In contrast, most bacteria employ a longer form of the hibernation promoting factor (HPF_long_) to stimulate 70S ribosomes dimerization (3, 5, 18, 24). The HPF_long_ proteins consist of the translational silencing N-terminal domain (NTD) and a dimerizing C-terminal domain (CTD) connected by an unstructured linker. Cryo-electron microscopy (cryo-EM) structures of the HPF_long_-bound 100S ribosomes from three *Firmicutes* (Staphylococcus aureus, Bacillus subtilis, and Lactococcus lactis) (25–28) have revealed a surprising mechanistic difference in 70S dimerization from that of the E. coli counterpart (29–32).

In *Firmicutes*, the CTD-HPF_long_ on one copy of the 70S ribosome directly interacts with another CTD-HPF_long_ that is tethered to the opposite copy of the 70S monomer, resulting in “back-to-back” conjoining of the two 30S subunits. There is no direct contact between hibernation factors in E. coli. Rather, the binding of RMF to the 30S subunits allosterically induces a “side-to-side” joining of 70S monomers at the 30S-30S interface. HPF_short_ binding further stabilizes the 100S complex. The structure and location of NTD-HPF_long_ and HPF_short_ are virtually superimposable, in that both occupy the tRNA- and mRNA-binding sites of the 30S subunits and thus sterically inhibit translation. RMF binds to a site that blocks the binding of the 30S subunit to the mRNA Shine-Dalgarno (SD) sequence. The physical occlusion of the ribosomal decoding sites and anti-SD region by these ribosome hibernation factors explains the repression of translation observed *in vivo* (10, 33) and *in vitro* (10, 15, 18), because the 100S pool likely titrates the functional ribosomes away from protein biosynthesis. The disassembly of the 100S dimers into ribosomal subunits, in principle, would provide a recyclable ribosome for a translational restart. We recently found that the GTPase HflX dissociates the S. aureus 100S ribosome in response to temperature upshift (34). In other bacteria, ribosome recycling factor (RRF) and initiation factor IF3 have been implicated in antagonizing 70S dimerization (35–37).

One of the outstanding questions about the 100S ribosome is the considerable variation in its temporal abundance across species. While the RMF-induced 100S ribosome accumulates only after transition to stationary phase, the firmicute 100S ribosome is continually produced throughout the life cycle (5, 17, 18, 33, 38, 39). These observations imply that the expression of *hpf*_long_ may be ill regulated. The significance of ribosome hibernation during exponential growth is completely unclear. In gammaproteobacteria and cyanobacteria, the hibernation factors appear to be more tightly regulated by small signaling molecules [cyclic AMP, (p)ppGpp, and polyamine] and stationary-phase-specific stressors (starvation and darkness) (40–42), whereas *hpf*_long_ is under positive transcriptional control of a general stress response alternate sigma factor SigB (3, 34). B. subtilis *hpf*_long_ is also subject to the sporulation sigma factor SigH (43) and ppGpp stringent response regulation (44). Unlike the closely related B. subtilis, we recently found that a S. aureus *sigB* knockout does not fully abolish *hpf*_long_ expression, and disrupting the major (p)ppGpp synthetase Rsh has no effect on HPF_long_ levels (34). These findings imply that additional regulators are involved and that the modulation of *hpf*_long_ expression is species specific.

**FIG 1:**
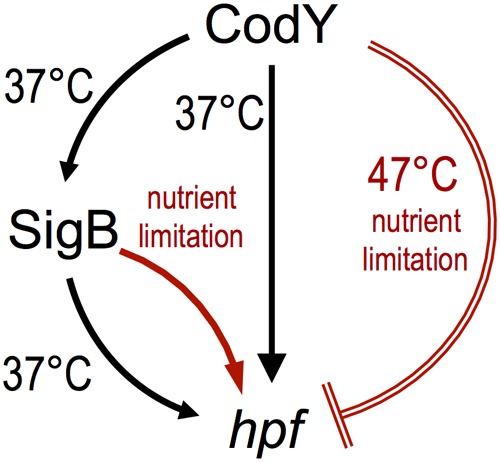
A proposed model of *hpf*_long_ regulation by CodY and SigB in S. aureus USA300 JE2. Under conditions that support growth, CodY positively controls *hpf_l_*_ong_ expression in SigB-dependent and SigB-independent fashions. Under certain suboptimal conditions, CodY represses *hpf*_long_ expression, whereas SigB upregulates *hpf*_long_ expression. Activation is shown as an arrow, and repression is shown as a crossbar. Unfavorable growth conditions are colored red.

In this study, we provide an explanation for the constant production of 100S ribosome in S. aureus USA300. We show that the transcription factor CodY plays a primary role in promoting S. aureus *hpf*_long_ expression and acts upstream of SigB in favorable environments, but it represses *hpf*_long_ under suboptimal conditions (Fig. 1). These regulatory phenomena appear to be strain specific. Furthermore, the pathophysiological significance of ribosome hibernation has not been fully examined despite the broad range of *in vitro* phenotypes. We demonstrate that perturbing the biogenesis and disassembly of the 100S ribosome negatively impacts the infectivity of S. aureus in a murine sepsis model. These results establish a new connection between ribosome preservation and pathogenesis, which is channeled through two master regulators (CodY and SigB) of virulence genes.

## RESULTS

### Disrupting the assembly and disassembly of hibernating 100S ribosomes severely attenuates S. aureus virulence.

**FIG 2:**
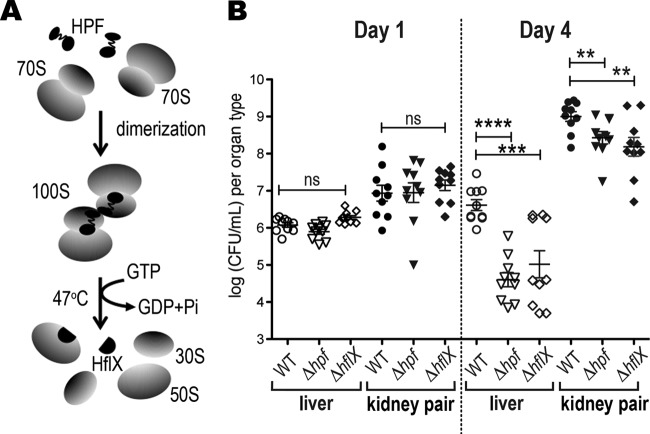
The Δ*hpf* and Δ*hflX* mutants are severely attenuated in a murine sepsis model. (A) An illustration of the opposing roles of HPF and HflX in 100S ribosome assembly and disassembly. (B) Bacterial burden in livers and kidneys 1 and 4 days postinoculation with the control PBS buffer, wild-type (WT) S. aureus, and its *hpf* and *hflX* knockouts (10 mice per treatment). Each kidney data point is generated from the kidney pair of the same animal. Less than 10 CFU was recovered from the PBS buffer control and not shown in the graph. Each data point is the mean value ± standard error (SEM). **, *P* < 0.01; ***, *P* < 0.005; ****, *P* < 0.001; ns, not significant by one-way ANOVA with Tukey’s test.

S. aureus is particularly adept at establishing persistent colonization in the host, which often leads to relapsing and recalcitrant infections. To gain insight into the role of hibernating ribosomes in staphylococcal pathogenesis, we evaluated the ability of the Δ*hpf* and Δ*hflX* mutants to replicate in a mouse model of sepsis (Fig. 2A). At 1 and 4 days after intravenous infection, we recovered S. aureus from the livers and kidneys and enumerated the CFU on tryptic soy broth (TSB) agar plates. S. aureus is halotolerant and normally thrives in kidneys. No significant differences were observed between the treatment groups in either organ type on day 1. By day 4, the bacterial burden in mice infected with the Δ*hpf* mutant decreased by two orders of magnitude in livers and 3-fold in kidneys relative to that in the wild type (WT). A similar downtrend was observed in the Δ*hflX* mutant, in which ∼30-fold and 5-fold fewer CFU counts were recovered from livers and kidneys, respectively (Fig. 2B). We previously showed that a 70S ribosome dimerizing mutant (Δ*hpf* mutant) loses 100S ribosome and cell viability in the long-term laboratory cultures and exhibits accelerated ribosome decay, heat susceptibility, and translational derepression, whereas a 100S ribosome disassociation mutant (Δ*hflX* mutant) displays thermotolerance and an accumulation of 100S ribosomes. These *in vitro* phenotypes were fully rescued by genetic complementation (10, 28, 34). Our animal study further strengthens these *in vitro* findings that an impaired metabolism of hibernating 100S ribosomes is disadvantageous for S. aureus infection.

### Expression of S. aureus *hpf*_long_ is regulated by CodY, and SigB and CodY modulation is strain dependent.

Constitutive expression of *hpf*_long_ contributes to the accumulation of 100S ribosomes throughout growth and post-stationary phase (10, 18, 24, 39). The regulation of *hpf*_long_ in all bacteria is not fully understood. The general stress response (GSR) sigma factor SigB is the major alternative sigma factor in S. aureus that controls the expression of ∼200 genes, many of which are virulence factors (45). We previously showed that *hpf*_long_ expression is partially compromised in a *sigB* mutant only under certain conditions (34), suggesting the involvement of another hitherto unknown regulator(s). A survey of multiple S. aureus genomes (http://aureowiki.med.uni-greifswald.de) (46) revealed two conserved minor alternative sigma factors (SigS_Sa_ and SigH_Sa_) as the potential candidates, in addition to a master virulence transcription factor CodY. CodY controls hundreds of metabolic and virulence genes in response to cellular GTP and nutrient availability (47). Unlike B. subtilis SigH that controls sporulation, S. aureus is not a sporeformer, and SigH_Sa_ has been linked to the expression of competence genes (48). SigS_Sa_ is an extracytoplasmic sigma factor whose expression is induced by cell wall and DNA-damaging agents (49). The spectrum of SigH_Sa_ and SigS_Sa_ regulons has not been fully explored.

**FIG 3:**
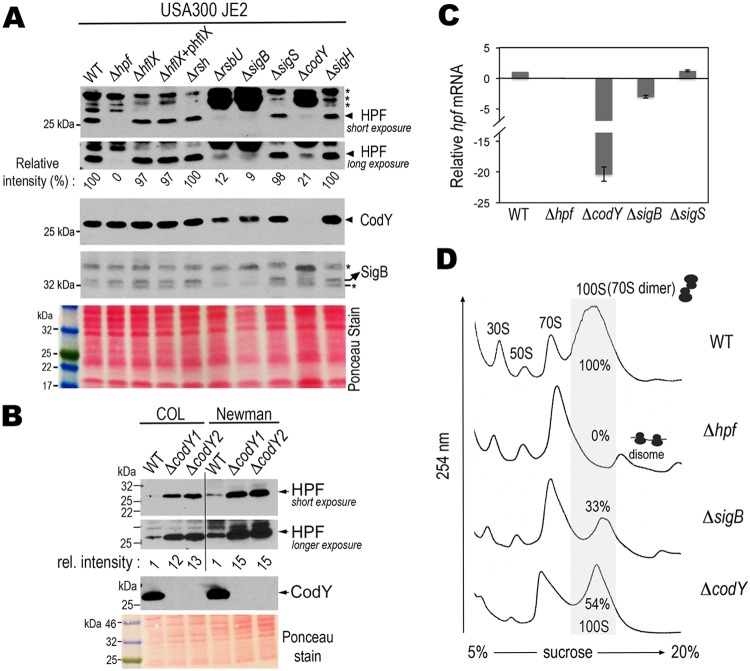
Strain-specific regulation of *hpf*_long_ expression by CodY and SigB in a nutrient-proficient environment. (A) CodY and SigB positively regulate *hpf*_long_ in S. aureus strain JE2. Western blots show the expression levels of HPF_long_, CodY, and SigB in different mutants grown in TSB (at a 3.75:1 tube-to-medium ratio) at 37°C until late log phase. Long and short exposures of the blots better present the relative intensities of HPF_long_ signals. Quantitation of the immunoblot signals was determined by ImageJ software and normalized to the wild-type (WT) signal. *, nonspecific cross-reaction bands. Ponceau red staining of the nitrocellulose membrane prior to antibody hybridization shows the total protein input. The data are representative of five independent repeats. (B) CodY negatively regulates *hpf*_long_ in S. aureus strains COL and Newman. Experiments were performed as in panel A. Two *codY* mutants from independent allelic-exchange mutagenesis were used to ensure reproducibility. (C) RT-qPCR demonstrates downregulation of the *hp*f_long_ mRNA in the strain JE2 Δ*codY* and Δ*sigB* mutants but not in the Δ*sigS* mutant. The housekeeping gene *polC* was used as an internal reference to obtain relative expression levels compared to that of the WT strain. Error bars are standard deviations from three independent biological samples. (D) Ribosome profiles verify the reduction of the 100S ribosome pool in *codY* and *sigB* knockouts. Crude ribosomes were prepared from cultures grown at a 5:1 flask-to-TSB ratio and were ultracentrifuged through a 5% to 20% sucrose gradient to separate different ribosomal complexes according to their mass. The amount of each ribosomal species is indicated by the absorbance at 254 nm on the *y* axis. ImageJ software was used to calculate the peak areas in the sucrose density gradient profiles and the amount of 100S ribosome was expressed in percentages relative to the WT. The profile is representative of three independent experiments.

We analyzed the amounts of HPF_long_ in the knockouts of the aforementioned regulatory genes under conditions that support rapid growth, in this case, 37°C in TSB. The regulators were chosen because they have previously been confirmed or implicated as important for virulence, stress tolerance, and long-term survival. These phenotypes are common features of an *hpf*_long_ mutant. Furthermore, conserved binding sites of some of these regulators, e.g., CodY and SigB, were bioinformatically identified within the *hpf* operon (see below). In line with our previous observation (34), a loss of the dissociation factor HflX and the major ppGpp synthetase Rsh does not affect HPF_long_ levels. The knockouts of *sigS* and *sigH* also did not exhibit measurable differences in HPF_long_ synthesis relative to that of the WT strain. HPF_long_ production was significantly reduced by at least 5-fold in *rsbU*, *sigB*, and *codY* mutants (Fig. 3A, top two panels). RsbU positively controls SigB activity by dephosphorylating the anti-anti-sigma factor RsbV and thereby releases SigB from its inhibitory complex with RsbW (50).

Previous transcriptomic studies in S. aureus strain UAMS-1 (pulsotype USA200, clonal complex 30 [CC30]) have shown that CodY negatively regulates *hpf*_long_ expression (51, 52). We were surprised to find that CodY has an opposite impact in our model strain JE2 (pulsotype USA300, CC8f). To test if the positive regulation by CodY is strain specific, we examined the production of HPF_long_ in the Δ*codY* mutants of two widely used strains, Newman and COL (both CC8a subclade). For S. aureus clonal lineages, see references 53 and 54. We found that *hpf*_long_ expression was derepressed in the COL Δ*codY* mutant and the Newman Δ*codY* mutant by 12- to 15-fold (Fig. 3B), in agreement with the results from strain UAMS-1. These variations suggest that differential regulation of *hpf*_long_ by CodY is strain specific.

We found that a JE2 Δ*codY* mutant also reduced *sigB* expression (Fig. 3A, fourth panel) and thus provided the first clue that SigB and CodY share an overlapping pathway. Reverse transcription-quantitative PCR (RT-qPCR) showed that the reduction of HPF_long_ protein levels was due to decreased *hpf*_long_ transcript levels (Fig. 3C). Although we cannot completely rule out the possibility of posttranscriptional mRNA turnover and protein degradation, the data suggest that the regulation of SigB and CodY primarily occurs at the transcriptional level. Finally, an analysis of the ribosome profile revealed that insufficiency of HPF_long_ in Δ*codY* and Δ*sigB* mutants reduced but did not fully abolish the formation of the 100S ribosomes (Fig. 3D).

### CodY-activated *hpf*_long_ expression masks the transcript from a SigB-dependent promoter.

**FIG 4:**
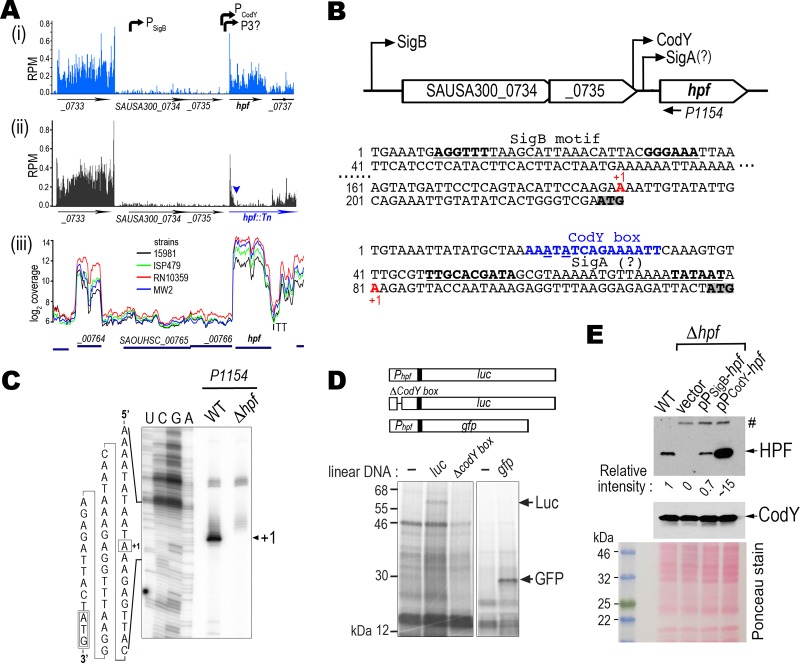
Identification of the promoters that drive *hpf*_long_ expression. (A) RNA-seq density profiles show the discrete transcriptional units of *hpf* operon. Panels i and iii show the high coverage of the *hpf*_long_ region in five S. aureus strains that suggests a separate transcriptional unit from the previously confirmed SigB promoter. Panel ii shows the density plot of a transposon insertion (triangle) of Δ*hpf* that abolishes the synthesis of *hpf*_long_. The trailing density after the insertion is the transposon transcript. RNA-seq results in panels i and ii were from two independent biological samples (10). RPM, reads per million reads. Panel iii was directly downloaded from the S. aureus transcriptome browser (http://staph.unavarra.es). (B) Relative location and sequence of SigB and CodY binding motifs (underlined). Core sequences are marked in boldface. Underlined blue letters indicate mismatches to the canonical motif. A putative SigA motif is marked with a question mark. Transcriptional start sites (+1) are labeled in red. Translational start codons of SAUSA300_0734 and *hpf* (locus SAUSA300_0736) are shaded. (C) Primer extension confirms the transcriptional start site (+1) preceding the *hpf*_long_. A radiolabeled ^32^P-P1154 antisense oligonucleotide (see its location in panel B) was used in the reverse transcription to map the 5′ end of the *hpf* transcript. The cDNA product was resolved on a 10% denaturing PAGE gel with sequencing lanes on the left. (D) *In vitro* coupled transcription-translation of *gfp* and *luc* reporters under the control of the CodY-dependent promoter programmed with S. aureus S-30 lysates. Protein products were identified by [^35^S]methionine incorporation. The disruption of the CodY box abolishes the synthesis of *luc*. (E) Both CodY-dependent and SigB-dependent promoters rescue the synthesis of *hpf*_long_ in a Δ*hpf* mutant. TSB cultures with a 3.75:1 tube-to-medium ratio were grown at 37°C until late log phase (OD_600_ of 1.4 to 1.6) and the cells were collected for immunoblotting. Empty plasmid pLI50 served as a negative control. Relative intensity of protein signals to the chromosomally encoded HPF_long_ (WT lane) was measured by ImageJ. #, nonspecific band derived from the pLI50. The figure represents one of the three independent repeats. Ponceau red stain shows the total protein load prior to antibody incubation.

Transcriptome sequencing (RNA-seq) data from our model strain and other S. aureus strains confirmed the architecture of the transcriptional unit (Fig. 4A). S. aureus *hpf*_long_ is the last gene in a three-gene operon (Fig. 4B). We found a perfectly matched SigB consensus sequence (AGGTTT[−35]-N_17_-GGGTAT[−10]) (55) located at the 5′ untranslated region (UTR) of the locus SAUSA300_0734 (Fig. 4B). We also observed relatively high read densities within the *hpf*_long_ region compared to that in the upstream loci, which suggests the existence of an additional transcriptional unit (Fig. 4A). An inspection of the 5′ region revealed a conserved CodY binding motif (AATTTTCWGAAAATT, where W is A/T) (52, 56). We performed a primer extension and mapped the transcriptional start site (TTS) of this second *hpf*_long_ transcript to an “A” that lies 38 nucleotides (nt) upstream from the *hpf*_long_ start codon (Fig. 4C). An imperfect sequence of a housekeeping SigA binding motif (TTGACA[−35]-N_17_-TGNTATAAT[−10]) (57) was detected immediately downstream of the CodY box (15 nt away from the −35 region). We found that a 140-bp 5′ UTR of *hpf*_long_ containing the CodY motif was sufficiently strong to drive the expression of heterologous luciferase (*luc*) and green fluorescent protein (*gfp*) genes in a cell-free coupled transcription-translation system, which was programmed with a linear DNA fragment of P_codY_-*luc* or P_codY_-*gfp* fusion and the S. aureus S-30 lysates. In the coupled transcription-translation reaction, the synthesis of reporters depends on the transcriptional activation of the reporter fusion DNA when the same S-30 extract is applied across all reactions. We surmised that the template DNA with a disrupted CodY motif would be unable to initiate transcription and thus translation would not occur. Indeed, a partial deletion of the CodY box abolished the synthesis of the *luc* reporter (Fig. 4D). These results support our speculation that the 5′ UTR of *hpf*_long_ constitutes an independent transcriptional unit and that CodY is the primary activator of *hpf*_long_ expression.

By attaching the individual CodY-dependent and SigB-dependent promoter regions directly to the *hpf*_long_ coding region (retaining the native *hpf*_long_ Shine-Dalgarno sequence) on a promoterless plasmid, our Western blot analysis showed that both promoters restored the expression of HPF_long_ in the Δ*hpf* mutant (Fig. 4E). The expression profile is consistent with the RNA-seq data (Fig. 4A) showing that CodY-dependent promoter is a much stronger promoter.

**FIG 5:**
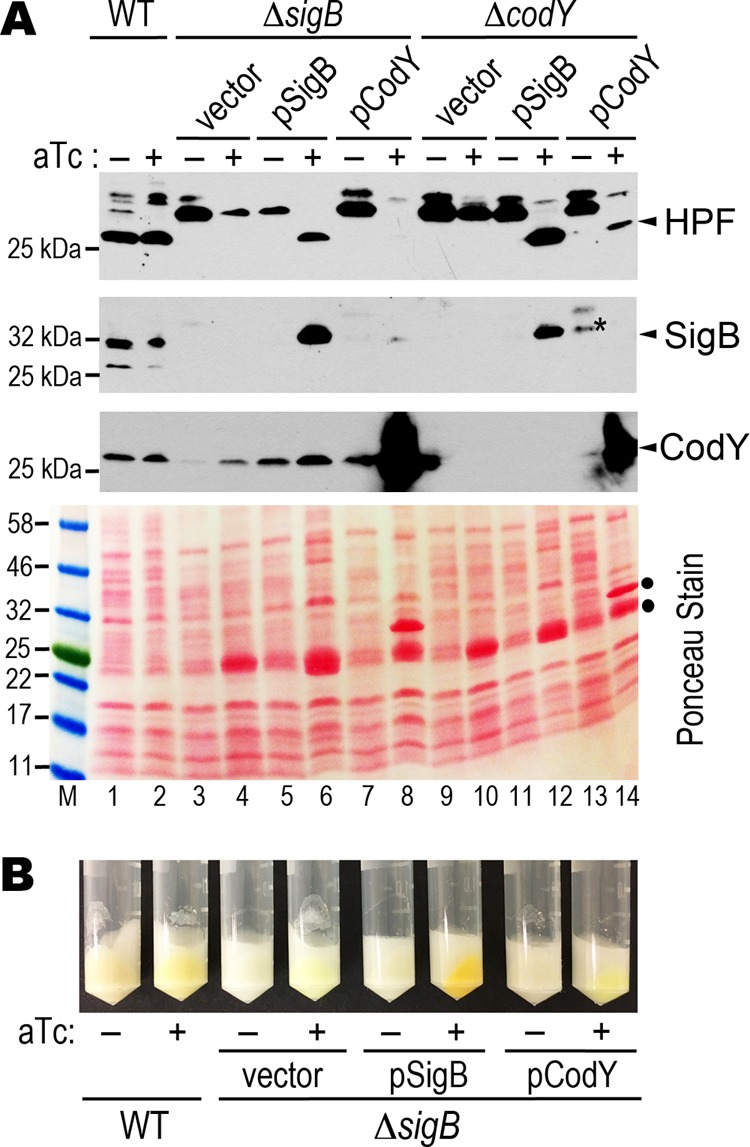
Expression of SigB rescues a Δ*codY* mutant. (A) Western blots showing the production of HPF_long_, SigB, and CodY in the Δ*codY* and Δ*sigB* mutants that are complemented with the pRMC2-borne SigB and CodY. A final 400 ng/ml of anhydrotetracycline (aTc) was added to TSB cultures (at a 3.75:1 tube-to-medium ratio; OD_600_ of ∼0.8) and induction was continued for an additional 2 h at 37°C. *, nonspecific band of anti-SigB that comigrates with the true SigB signal. Ponceau red staining of the nitrocellulose membrane prior to antibody hybridization shows the total protein input. •, overexpression of the plasmid-borne products upon aTc induction. (B) Restoration of the production of staphyloxanthin (orange pigment) in the Δ*sigB* mutant complemented with a SigB plasmid but not with a CodY plasmid. Bright yellowish pellets are due to the color of aTc.

To assess the relationship between SigB and CodY, we complemented the Δ*sigB* mutant with CodY and SigB expressed on the pRMC2 plasmid under the control of an anhydrotetracycline (aTc)-inducible promoter. Conversely, we attempted to rescue the Δ*codY* mutant with the same plasmids. aTc tightly controlled the expression of SigB and CodY, because no proteins were detected in the absence of the inducer (Fig. 5A). A restoration of HPF_long_ synthesis would indicate a successful complementation. From the immunoblot analyses, we found that a plasmid-encoded CodY only complemented a Δ*codY* mutant (Fig. 5A, lane 14), but a plasmid-encoded SigB complemented both the Δ*sigB* and Δ*codY* mutants (Fig. 5A, lanes 6 and 12). SigB regulates the production of the S. aureus orange carotenoid staphyloxanthin, and a *sigB* deletion mutant is white (58). We found that providing the *sigB*, but not *codY*, in *trans* fully rescued the pigmentation of the Δ*sigB* mutant upon aTc induction (Fig. 5B). From these results, we conclude that SigB acts downstream of CodY in *hpf*_long_ regulation. We noted that pigmentation was unaffected in a Δ*codY* mutant, suggesting that the biosynthesis of staphyloxanthin does not follow a CodY-to-SigB pathway.

### CodY is a repressor of *hpf*_long_ under thermal stress.

**FIG 6:**
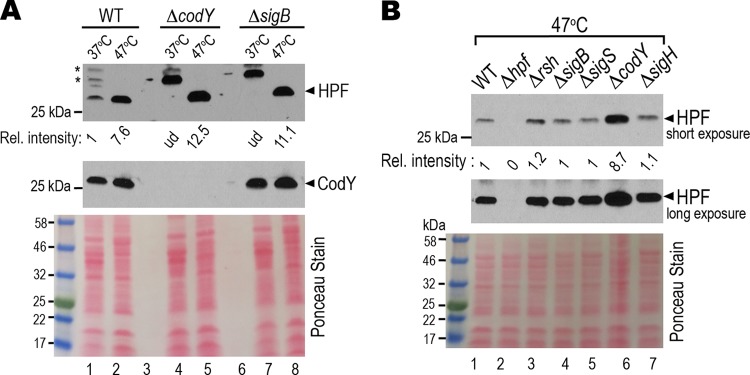
CodY negatively regulates *hpf*_long_ under heat stress, whereas SigB has no detectable impact on *hpf*_long_ expression. (A) HPF_long_ is overexpressed under thermal stress. Side-by-side comparison of the HPF_long_ synthesis in TSB cultures (at a 3.75:1 tube-to-medium ratio) at 37°C and 47°C by Western blot analysis. 37°C grown TSB cultures were collected at late log phase (OD_600_ of ∼1.4). Cells from 47°C TSB cultures were harvested 2.5 h postinoculation (OD_600_ of ∼0.25) because cell viability dramatically dropped beyond 3 h. *, nonspecific cross-reaction signals. Ponceau red-stained membrane shows equal protein load. Signal intensity was quantitated by ImageJ and calculated relative to that of the WT strain. The blot represents one of the four independent repeats. ud, undetectable under the given exposure. (B) CodY represses *hpf*_long_ expression at 47°C. Western blotting was performed as in panel A but with different genetic backgrounds.

We previously found that a loss of 100S ribosomes renders S. aureus susceptible to heat (28). We reasoned that 70S ribosome dimerization protects the ribosome from thermal damage and that the expression of *hpf*_long_ is heat-inducible. By comparing the HPF_long_ synthesis at 37°C and 47°C using equal amounts of protein input on the Western blots, we confirmed that the expression of HPF_long_ was upregulated by approximately 7-fold at 47°C (Fig. 6A, lanes 1 and 2). In striking contrast to the downregulation of *hpf_l_*_ong_ expression in the Δ*codY* background at 37°C (Fig. 3A), the synthesis of HPF_long_ was derepressed in the Δ*codY* mutant at 47°C (Fig. 6A, lanes 4 to 5; Fig. 6B, lane 6). Unlike the downregulation observed at 37°C (Fig. 3A), HPF_long_ expression was unaffected by *sigB* mutation at 47°C (Fig. 6A, lanes 7 to 8; Fig. 6B, lane 4). In contrast, the nonregulators SigS and SigH did not impact *hpf*_long_ expression at either temperature (Fig. 6B, lanes 5 and 7). These results demonstrate that CodY functions both as an activator (at 37°C) and a repressor (at 47°C) of *hpf*_long_, but positive SigB-mediated regulation occurs only under specific conditions, e.g., 37°C and nutrient limitation (see below).

### HPF_long_ expression is subject to changes in nutritional status.

**FIG 7:**
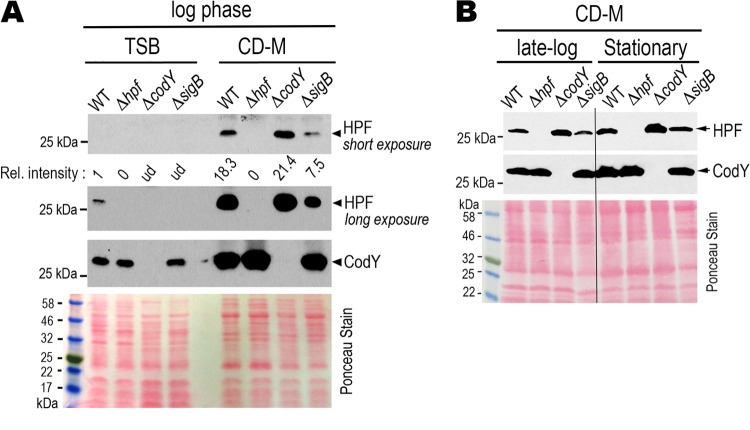
CodY and SigB have a negative and a positive role on *hpf*_long_ expression, respectively, in chemically defined medium (CD-M). (A) Comparison of HPF levels in TSB and CD-M. Cells were grown in TSB or CD-M (at a 3.75:1 tube-to-medium ratio) at 37°C and harvested during late log phase (TSB, OD_600_ of 1.4) and log phase (CD-M, OD_600_ of 0.3). Western blots showing greater production of HPF_long_ in nutrient-rich TSB relative to that in the CD-M. A Δ*codY* mutant has increased *hpf*_long_ synthesis, whereas the Δ*sigB* mutant had moderately decreased synthesis. The figure is representative of three independent replicates. Relative intensity of the signals was quantitated by ImageJ. Ponceau red stain prior to antibody hybridization shows the total protein input. ud, undetectable. (B) CodY-mediated negative regulation of *hpf*_long_ continues through late log (OD_600_ of 0.8) and stationary (OD_600_ of 1.2) phases in CD-M. With the exception of the Δ*sigB* mutant (maximum OD_600_ of ∼0.7), all cells reached the desired OD_600_ when grown in 37°C CD-M at a 3.75:1 tube-to-medium ratio. The result is representative of two independent repeats. Total protein input was monitored by Ponceau red staining prior to the antibody incubation.

The DNA binding activity of CodY is greatly influenced by GTP concentration and nutrient availability (51, 59). We compared the *hpf*_long_ expression between the routinely used TSB and a chemically defined Pattee-Neveln medium (CD-M) (60). We found that HPF_long_ was strongly induced in the CD-M whereas with the same total protein input HPF_long_ was barely detectable in TSB during logarithmic growth (Fig. 7A, WT lanes). Similar to the 47°C TSB culture but to a lesser extent, HPF_long_ expression was moderately derepressed in the Δ*codY* CD-M culture. This mild derepression continued upon entry into stationary phase (Fig. 7B). Following the same negative trend as the Δ*sigB* mutant grown in TSB (Fig. 3A), SigB was required for full expression of HPF_long_ in CD-M culture (Fig. 7A). These findings confirm that *hpf*_long_ expression is sensitive to nutritional cues that are recognized by CodY and SigB.

## DISCUSSION

The means by which S. aureus maintains a large quantity of hibernating 100S ribosomes throughout its life cycle has been puzzling. The previous identification of SigB as the positive regulator of the dimerizing factor HPF does not fully explain how *hpf* is modulated under conditions outside SigB control. Here, we show that CodY is one of the missing links. CodY coordinates with SigB to ensure that HPF is sufficiently produced to generate 100S ribosomes in diverse environments. We show that the formation and timely dissociation of 100S ribosomes are necessary for S. aureus infection. Consistent with our results, *hpf* mRNA levels were induced by >25-fold during the infection period (61). CodY regulates a repertoire of metabolic genes, exoproteins, and genes involved in motility, competence, and the uptake of sugar, peptides, and iron (47, 62–65). Our finding that CodY modulates ribosome hibernation adds to a growing list of CodY-controlled cellular pathways.

A loss of *codY* or *sigB* significantly reduced the production of HPF_long_ but did not completely abolish the formation of 100S ribosomes (Fig. 3C). Many ribosome-binding proteins, despite their low cellular concentrations (≥10-fold ribosome over ligand), can be recycled for multiple rounds of association and dissociation, e.g., initiation factors, elongation factor EF-P, and release factor 1 (RF1) (66–68). By analogy, a small fraction of HPF_long_ in the *codY* and *sigB* mutants may account for the maintenance of a subpool of 100S ribosomes.

We found that the CodY-regulated expression of HPF_long_ is strain dependent (Fig. 3A and B). CodY of strain USA300 JE2 positively modulates *hpf*_long_, whereas it represses *hpf*_long_ in strains COL and Newman. These strain-specific variations are not unique for *hpf*_long_ but instead are common in S. aureus due to mutations in the regulatory genes and differences in stress response and metabolic capabilities (69, 70). For example, a nonsense mutation in the positive regulator (*rsbU*) of SigB, a truncation of TcaR transcription factor, and the instability of *agr* RNA have been observed in different strains. Most of the routinely used S. aureus strains, including Newman, UAMS-1, COL, and USA300, are defective in at least one regulatory or global sensory pathway (69).

The activity of CodY is strongly influenced by the nutritional status. Previous studies have shown that S. aureus USA300 has a much higher capacity to metabolize a wider range of carbohydrates and amino acids than the strains COL, Newman, and UAMS-1 due to nonsynonymous substitutions in the metabolic genes (54, 70). These metabolic differences might directly or indirectly influence CodY activity on its target DNA, resulting in the opposing roles of CodY observed in different genetic backgrounds.

The binding of GTP (in some bacteria) and branched-chain amino acids (BCAA) to CodY enhances its affinity to the target DNAs that carry a 15-nt palindromic sequence (56). Many true direct targets of CodY, however, do not strictly adhere to this rule and instead can tolerate up to four mismatches (56, 64). Although the CodY box of *hpf* has two mismatches, our cell-free transcription-translation data confirm that it is functional (Fig. 4D). A similar CodY box upstream of B. subtilis *yvyD* (homolog of *hpf*) has been identified by a genome-wide DNA-binding sequencing approach (56). The fact that the CodY motif is also proximal to the downstream RNA polymerase (RNAP) binding sites (Fig. 4B) (71) reinforces the premise that *hpf* is the direct target of CodY. In contrast, positive regulation of CodY on *sigB* is likely to be indirect. We were unable to find a CodY-like motif in the entire *sigB* operon, despite lowering the sequence stringency. In S. aureus, three promoters have been experimentally verified in the *sigB* operon. *sigB* undergoes positive autoregulation by controlling the transcription of *rsbV-rsbW-sigB* (50). This is distinct from the L. monocytogenes CodY that physically interacts with a region upstream of the *rsbV* region and represses the synthesis of *rsbV-rsbW-sigB* (64). The difference is not surprising, because the systems regulating SigB vary considerably among Staphylococcus, Listeria, and Bacillus species (45).

CodY primarily serves as a repressor of target genes and only acts as an activator of a limited number of targets (64, 72). We found that *hpf* is a member of the rare positive regulon during rapid exponential growth (Fig. 3). CodY may exert its positive effect by either stabilizing the binding of RNAP, altering the DNA structure to promote DNA melting, or mutually excluding the binding of a negative regulator. Conversely, CodY negatively regulates *hpf* under stress conditions (Fig. 6 and 7) due to the interference of RNAP binding to the promoter or competition with a positive regulator. Our findings that CodY can switch between two opposing roles on *hpf* strongly support the notion that CodY is a “molecular shifter,” whose physical action on DNA with respect to the RNAP and potentially another regulator(s) is still incompletely understood. Furthermore, it is possible that other small molecule ligands beyond the known GTP and BCAA participate in the role reversal of CodY. These effectors may compete with GTP or BCAA for CodY binding and thereby alter the oligomeric state and binding affinity of CodY. Differences in ligand selectivity have been observed. For instance, CodY proteins from Streptococcus pneumoniae and Lactococcus lactis do not bind GTP (59).

Transcriptional regulation is probably not the only way to regulate *hpf* concentration. Posttranscriptional regulation and protein turnover of ribosome hibernation factors provide additional layers of control over 100S ribosome abundance. E. coli *rmf* has an unusually long-lived transcript that lasts for hours compared to the average E. coli transcripts with a half-life of 1 to 2 min (73). S. aureus HPF protein is stable in culture after 4 days even when the ribosome concentration drops substantially (10). In Pseudomonas aeruginosa, the mRNA structure of the 5′ UTR and a portion of the *hpf* coding region appear to govern the translational efficiency of *hpf* (74). The 5′ UTR of Vibrio cholera *yfiA* is a target of an inhibitory small RNA VrrA, resulting in the downregulation of *yfiA* and upregulation of *hpf*, whose gene products compete for the common binding site on ribosomes (14). B. subtilis *hpf* is activated by both SigH and SigB (3), the stringent response alarmone (44), and most likely also by CodY (56). In this study, we show that the expression of S. aureus *hpf* is insensitive to *rsh* knockout and instead is differentially relayed through the SigB and CodY pathways in nutrient- and temperature-dependent manners. Therefore, the distinct regulators employed by two closely related species may have evolved semi-independently to facilitate bacterial adaptation under conditions encountered in their specialized niches.

## MATERIALS AND METHODS

### Bacterial strains and culture conditions.

Methicillin-resistant Staphylococcus aureus (MRSA) USA300 strain JE2 (GenBank CP000255) was used throughout the study. The construction of the Δ*hpf* (gene locus SAUSA300_0736), Δ*hflX* (SAUSA300_1198), Δ*rsh* (SAUSA300_1590), and Δ*sigB* (SAUSA300_2022) mutants has been described previously (10, 34, 39). *Bursa aurealis* transposon insertion mutants of S. aureus *rsbU* (SAUSA300_2025), *sigS* (SAUSA300_1722), c*odY* (locus SAUSA300_1148), and *sigH* (locus SAUSA300_0519) were obtained from BEI Resources and confirmed by PCR (75). The strains Newman and COL were generously provided by Anthony Richardson (University of Pittsburgh). The mutant alleles were subsequently introduced into a clean background by ϕ11 (for JE2) and ϕ80 (for Newman and COL) phage transduction. S. aureus strains were routinely grown in tryptic soy broth (TSB; Difco) or chemically defined Pattee-Neveln medium (CD-M) (60). TSB cultures at 47°C were harvested at 2.5 h postinoculation. Total mRNA, protein lysates, and crude ribosomes were prepared from late-log-phase TSB cultures. CD-M cultures were collected from log phase, late log phase, and overnight growth at 37°C. When necessary, erythromycin, chloramphenicol, and anhydrotetracycline (all from Sigma-Aldrich) were used at 5 μg/ml, 10 μg/ml, and 400 ng/ml, respectively. Cells were routinely grown in 15-ml culture tubes at a 3.75:1 tube-to-medium ratio and with an initial inoculum at an optical density at 600 nm (OD_600_) of 0.08. In larger cultures necessary for ribosome isolation, 50-ml TSB cultures were grown in a 250-ml flask at a 5:1 flask-to-medium ratio with 1:100 dilutions of the overnight seed cultures.

The primer sequences are listed in Table 1. S. aureus shuttle vectors pLI50 (76) and pRMC2 (77) were used for cloning and genetic complementation. The *sigB* coding region was PCR amplified with P1152 and P1153, whereas *codY* coding was PCR amplified with P1149 and P1125 using JE2 genomic DNA as the template and subsequently cloned into the KpnI and EcoRI sites of pRMC2 under the control of an anhydrotetracycline-inducible promoter. The CodY-dependent promoters linked to the green fluorescent protein gene (*gfp*) and luciferase gene (*luc*) were PCR amplified from plasmids pPROBE-gfp (78) and pBESTluc (Promega) by two-step crossover PCR (79) using primer pairs P651/P751 and P750/P756 and primer pairs P651/P749 and P748/P649, respectively. The *gfp* and *luc* DNA fragments were cloned into the BamHI/HindIII and BamHI/XbaI sites of pLI50, respectively, yielding pLI50gfp and pLI50luc. The deletion of the CodY box on pLI50gfp was introduced with a QuikChange mutagenesis kit (Agilent Genomics). The construction of the plasmid pP_codY_-*hpf* (formerly pLI50hpf) was reported previously (10, 39). To construct a SigB-dependent promoter fusion of *hpf* (pP_sigB_-*hpf*), a two-step PCR using primer pairs P1197/P1198 and P1199/P627 was used to amplify the P_sigB_-*hpf* fragment from the JE2 DNA template. The PCR product was ligated into the BamHI/HindIII sites of pLI50. The pLI50 and pRMC2 derivatives were passaged through a restriction-deficient S. aureus RN4220, reisolated, and electrotransformed into the destination backgrounds.

**TABLE 1:** Primers used in this study

Application or target	Primer	Sequence (5′→3′)^*a*^
*sigB* coding region	P1152	ATCTGGTACCACAATCAGTATGACTAAGTATATAA
	P1153	TAGAATTCAAATTCTATTGATGTGCTGCTTCTTGTAATTTCT
*codY* coding region	P1149	ACAGGTACCGATTTAAGTGCATTTATTCTATA
	P1125	CAGAGAATTCGACTTATTTACTTTTTTCTAATTCATC
P_codY_-*luc*	P651	CGGGATCCATACAACTGGATTAACAATTCATCGTGCAGGGTG
	P749	CTTTATGTTTTTGGCGTCTTCCATAGTAATCTCTCCTTAAACCTCTTTAT
	P748	ATAAAGAGGTTTAAGGAGAGATTACTATGGAAGACGCCAAAAACATAAAG
	P649	ATTCTAGACTATTACAATTTGGACTTTCCGCCCTT
P_codY_-*gfp*	P651	CGGGATCCATACAACTGGATTAACAATTCATCGTGCAGGGTG
	P751	TGAAAAGTTCTTCTCCTTTACTCATAGTAATCTCTCCTTAAACCTCTTTATATAAAGAGGTTTAAGGAGAGATTAATA
	P750	AAGAGGTTTAAGGAGAGATTACTATGAGTAAAGGAGAAGAACTTTTCA
	P756	TGTCTAGATTTCTTGTTTATTTATTCAAGACCGACTTTTTTGCGGT
P_sigB_-*hpf*	P1197	TTAGGATCCGGTGGATTAGGTTTAGGCTATG
	P1198	TCTAATCATAGTAATCTCTCCTTATCGACCCAGTGATATACAATTTCTG
	P1199	CAGAAATTGTATATCACTGGGTCGATAAGGAGAGATTACTATGATTAGA
	P627	TGAAGCTTTAAACTTAATTTATTGTTCACTAGTTTGAATCAAGCC
Sequencing primers on pLI50 MCS	P630	GCACATTTCCCCGAAAAGTGCCACCTGACGT
	P631	TGCCTTTATTTTGAATTTTAAGGGGCAT
Primer extension, *hpf*	P1154	ACTTTAACATGCGCCACTGCATTTGGT
Sequencing primers on pRMC2 MCS	P212	GATAGAGTTATTTGTCAAACTAG
	P213	CAAGGCGATTAAGTTGGG
qPCR, *polC*	P1205	CAGGTGACACAGCGGGTATA
	P1206	TGCCGGGTTGTGATGCTATT
qPCR, *hpf*	P887	TGGATTCAGAAGAAGCGGTATT
	P888	TACGGCGGTAAACGATACTTG
QuikChange deletion of CodY motif (ΔCAGAAAA)	P1193	ATTATATGCTAAAAATATTTCAAAGTGTTTGCGTTT
	P1194	AAACGCAAACACTTTGAAATATTTTTAGCATATAAT

a Restriction enzyme recognition sites are underlined.

### Animal studies.

All animal experiments were approved by the Saint Louis University Institutional Animal Care and Use Committee (protocol 2640, PHS assurance number A-3225-01). Saint Louis University is an AAALAC-accredited institution and adheres to the standards set by the Animal Welfare Act and the NIH *Guide for the Care and Use of Laboratory Animals*.

Six-week-old female C57BL/J mice averaging 17.2 ± 0.9 g (Jackson Laboratory) were intravenously injected with either 100 μl of phosphate-buffered saline (PBS), or 100 μl of 4 × 10^6^ CFU of S. aureus strains. On day 1 or day 4 postinfection, the mice were euthanized. Mouse livers and kidneys were removed, homogenized in 1 ml of sterile PBS in a closed system tissue grinder (SKS Science), and dilution plated on TSB agar plates to enumerate CFU after 24 h of incubation at 37°C. Statistical significance was determined with one-way analyses of variance (ANOVAs). Tukey’s multiple-comparison tests were performed after ANOVAs with GraphPad Prism, version 7, to analyze the differences in the effects of each treatment.

### Mapping of the transcriptional start site.

A total of 4 μg of total RNA was used to map the *hpf* transcriptional start site. Primer extension (80) was carried out at 37°C for 1 h using a [γ-^32^P]ATP-labeled oligonucleotide that complemented a region ∼100 nt downstream of the potential transcription start site. The resulting cDNA was extracted once with phenol-chloroform (pH 6.8; Amresco) and was finally precipitated using 0.3 M sodium acetate (pH 5.2) and 3 volumes of isopropanol relative to the original reaction volume. The DNA pellet was washed with 70% ethanol, and the air-dried pellet was resuspended in 5 μl of formamide-containing loading buffer. DNA sequencing ladders were generated using the USB Thermo SEQ kit (Affymetrix). Primer extension products and 1 μl of ladders were heat denatured and resolved on 10% Tris-borate-EDTA (TBE)-urea polyacrylamide (29:1) sequencing gels and scanned on a GE Typhoon phosphorimager.

### *In vitro* coupled transcription-translation.

S-30 extracts were prepared from S. aureus JE2 by cryomilling cell disruption (see “Ribosome profile analysis” below). A runoff reaction was performed by incubating the lysate at 25°C for 70 min with 0.15 volume of runoff premix (0.75 M HEPES [pH 7.5], 7.5 mM dithiothreitol [DTT], 21.3 mM magnesium acetate, 75 μM twenty l-amino acids, 6 mM ATP, 20 mg/ml phosphoenolpyruvate, 50 U pyruvate kinase) relative to the volume of lysate input. The extracts were then dialyzed in Slide-A-Lyzer cassettes (Thermo Fisher) against three changes of buffer A (20 mM HEPES [pH 7.5], 14 mM magnesium acetate, 100 mM potassium acetate, 1 mM DTT, 0.5 mM phenylmethylsulfonyl fluoride [PMSF]), centrifuged at 4°C at 20,800 × *g* for 10 min, and stored at −80°C.

Linear DNA fragments containing the *hpf* promoter fused to a *gfp* or a *luc* reporter were PCR amplified with P630/P631 (Table 1) using pLI50gfp or pLI50luc as a template. Typical 25-μl reaction mixtures contained 500 ng of DNA template, 10 μl of translation premix (81), 2.5 μl of 1 mM l-amino acids lacking methionine, 7.5 μl of S-30 extract, 200 ng/μl anti-*ssrA* oligonucleotide (5′-TTAAGCTGCTAAAGCGTAGTTTTCGTCGTTTGCGAGTA-3′), and 10 μCi Tran^35^S-label (MP Biomedicals). After a 1-h incubation at 37°C, protein samples were precipitated in 4 volumes of acetone, resolved on 4% to 20% TGX SDS-PAGE gels (Bio-Rad), and autoradiographed.

### Western blots.

S. aureus cell pellets were homogenized with Lysing matrix B (MP Biomedicals, 100 mg beads/ml cells) in 25 mM Tris (pH 7.5) on a Retsch MM400 mixer mill at 15 Hz in four 3-min cycles. Clarified lysates were recovered by spinning at 20,817 × *g* at 4°C for 5 min to remove cell debris. A total of 0.1 to 0.2 *A*_280_ unit of cell lysate were analyzed on 4% to 20% TGX SDS-PAGE gels (Bio-Rad), and the proteins were transferred to a nitrocellulose membrane using a Trans-Blot Turbo system (Bio-Rad). The membrane was stained with Ponceau red (Amresco) to ensure equal loading, followed by immunoblotting using a 1:6,000 dilution of anti-HPF (39), a 1:1,000 dilution of anti-SigB (a gift from Markus Bischoff), and a 1:20,000 dilution of anti-CodY (ETU005; Kerafast). To detect multiple protein targets, the same membrane was stripped with the Restore Western blot stripping buffer (Thermo Fisher) and reprobed with the desired antibody. The intensity of immunoblot bands was quantitated by ImageJ.

### Ribosome profile analysis.

Cell pellets from a 50-ml late-log-phase TSB culture (OD_600_ of ∼1.4) were resuspended in buffer B (20 mM HEPES [pH 7.5], 14 mM MgCl_2_, 100 mM KCl, 0.5 mM PMSF, 1 mM DTT) and fresh frozen in liquid nitrogen. Crude ribosomes were extracted from frozen pellets by pulverizing on a cryomiller (Retch MM400) using four 3-min cycles at 15 Hz in 10-ml grinding jars with a 15-mm grinding ball. The resulting milled cells were thawed in a 30°C water bath for 5 to 8 min and then immediately placed in an ice bath for 10 min. The lysate was centrifuged at 20,000 × *g* for 10 min at 4°C. The clarified lysate was recovered and spun at 20,817 × *g* at 4°C for 5 min to remove residual debris. Five *A*_260_ units of RNA was layered on a 5% to 20% (wt/vol) sucrose density gradient made in buffer B (20 mM HEPES [pH 7.5], 10 mM MgCl_2_, 100 mM NH_4_Cl) that was equilibrated with a BioComp Gradient Master. The gradients were centrifuged at 210,000 × *g* at 4°C in an SW41 rotor for 2.5 h. Fractionation was performed using a Brandel fractionation system equipped with a UA-6 UV-visible detector.

### Reverse transcription-quantitative PCR.

Total RNA was extracted using a modified hot phenol-SDS method (82) and an RNeasy kit (Qiagen). DNA contaminants were removed using two successive digestions with Turbo DNase I (Ambion), and RNA integrity was verified by nondenaturing agarose gel electrophoresis and ethidium bromide staining (83). Intact RNA was judged by the relative intensity of 23S and 16S rRNA bands with a minimum accepted ratio of 1:1. RT-qPCR was performed essentially as described previously (84). Briefly, first-strand cDNA synthesis was performed with 5× iScript Supermix (Bio-Rad) and 50 ng/μl of DNase I-treated RNA. Quantitative PCR was performed in triplicates in 20-μl reaction mixtures containing 1× iTaq Universal SYBR green supermix (Bio-Rad), 0.4 μM primers (Table 1), and 2 μl of cDNA on a CFX96 real-time PCR instrument (Bio-Rad). The DNA polymerase III gene (*polC*) was used as an internal reference (51). Differences in mRNA levels were calculated using a published 2^−ΔΔ^*^CT^* formula (85).

### *In silico* analyses.

Total mRNA-seq of S. aureus JE2 was extracted from our previous ribosome profiling project (10), under NCBI GEO accession GSE74197. The read densities were processed as reads per million reads (RPM) and were visualized in MochiView (86). Transcriptomics data of other strains were taken directly from the S. aureus transcriptome browser (http://staph.unavarra.es) (87).
